# PTHrP-related Hypercalcaemia in Infancy and Congenital Anomalies of the Kidney and Urinary Tract (CAKUT)

**DOI:** 10.1186/s40697-015-0052-y

**Published:** 2015-05-11

**Authors:** Nardeen Kodous, Guido Filler, Ajay Parkash Sharma, Tamara Angela Van Hooren

**Affiliations:** Department of Paediatrics, Division of Nephrology, University of Western Ontario, 800 Commissioners Road East, Rm B1-436, London, ON N6A 5 W9 Canada; Department of Paediatrics, Division of General Paediatrics, University of Western Ontario, London, ON Canada

**Keywords:** Hypercalcemia, Treatment, Infant, Failure to thrive, Chronic kidney disease

## Abstract

**Background:**

Severe hypercalcaemia is a rare but clinically significant condition in infancy and childhood. Parathyroid hormone-related peptide (PTHrP)-mediated hypercalcaemia resulting from a malignancy is rare and only a handful of case reports have outlined its incidence alongside a benign condition.

**Objectives:**

To describe the diagnostic workup and management of an infant with hypercalcaemia, renal dysplasia, and elevated PTHrP levels.

**Design:**

Case report.

**Setting:**

The Victoria Hospital campus of the London Health Sciences Centre in London, Ontario, Canada.

**Patients:**

A child with congenital anomalies of the kidneys and urinary tract (CAKUT), stage 2 chronic kidney disease (CKD), and renal dysplasia who presented with severe hypercalcaemia.

**Measurements:**

Weight, renal ultrasound, creatinine, cystatin C, eGFR, calcium, urea, bicarbonate, serum sodium, fractional sodium excretion, urine calcium to creatinine ratio, PTH, TSH, Free T4, AM cortisol, HMA, VMA, 25-vitamin D, 1,25 dihydroxy-vitamin D, calcitriol, vitamin A, ACE levels, skull and chest x-rays, alkaline phosphatase, CBC, tumour lysis profile, catecholamine breakdown, whole-body MRI, PTHrP.

**Methods:**

Full diagnostic workup and patient management. Patient treated with intravenous hydration, furosemide, calcitonin and CalciLo.

**Results:**

PTHrP was elevated and no evidence of a malignancy was found. Treatment consisting of a low-calcium CalciLo diet (in place of breast milk) adequately controlled the patient’s hypercalcaemia. Hypercalcaemia associated with CAKUT in infancy is not all that uncommon and was reported in 15/99 infants in another study, most of whom had a suppressed PTH similar to that of our patient. PTHrP was not measured in these cases and may have also been elevated.

**Limitations:**

The study is limited in that it is a description of a single patient case. Future measurement of PTHrP in similar patients is necessary to confirm our results.

**Conclusions:**

The possibility of elevated PTHrP levels must be considered in patients with known renal dysplasia who are differentially diagnosed with hypercalcaemia.

## Abrégé

**Contexte:**

L’hypercalcémie aigue est rare, bien qu’elle constitue un trouble important de la petite enfance et de l’enfance du point de vue clinique. Rares sont les hypercalcémies à médiation de peptide lié à l’hormone parathyroïdienne (PTHrP) qui résultent d’une malignité, et seules quelques observations cliniques témoignent de leur incidence dans les cas de troubles bénins.

**Objectifs:**

Décrire le bilan diagnostic et la prise en charge d’un petit enfant atteint d’hypercalcémie, de dysplasie rénale, et présentant des taux élevés de PTHrP.

**Type d’étude:**

Observation clinique.

**Contexte:**

Le campus de l’Hôpital Victoria du Centre de services de santé de London (London Health Sciences Centre), à London, en Ontario, au Canada.

**Participants:**

Un enfant atteint d’anomalies congénitales du rein et des voies urinaires (CAKUT), d’insuffisance rénale chronique (IRC) de stade 2 et de dysplasie rénale, qui présentait une hypercalcémie aigue.

**Mesures:**

Le poids, l’échographie rénale, la créatinine, la cystatine C, le R-EGF, le calcium, l’urée, le bicarbonate, le sodium sérique, la fraction d’excrétion du sodium, le ratio calcium/créatine dans l’urine, la PTH, la TSH, le T4 libre, le taux de cortisol le matin, l’acide homomandelique, l’acide vanillylmandelique , le 25-vitamine D, le 1,25 dihydroxy-vitamine D, le calcitriol, la vitamineA, les taux d’enzyme de conversion de l’angiotensine, les radiographies thoraciques et crâniennes, la phosphatase alcaline, la formule sanguine complète, le profil de lyse tumorale, les taux de catécholamine, la résonnance magnétique du corps entier, le taux de PTHrP

**Méthodes:**

Bilan diagnostic et prise en charge du patient. Le patient a reçu une hydratation intraveineuse, du furosémide, de la calcitonine et du Calcilo.

**Résultats:**

Le taux de PTHrP était élevé et on n’a découvert aucune trace d’une malignité. Le traitement, qui consistait en un régime faible en calcium de Calcilo (en remplacement du lait maternel), a permis de limiter adéquatement l’hypercalcémie du patient. L’hypercalcémie associée à des anomalies congénitales du rein et des voies urinaires dans la petite enfance n’est pas si rare. Une autre étude a fait état de 15/99 petits enfants, dont la plupart présentait un faible taux de PTH semblable à celui de notre patient. Le PTHrP, qui n’a pas été mesuré dans ces cas, pourrait également avoir été élevé.

**Limites de l’étude:**

L’étude est limitée en ceci qu’elle consiste en la description d’un seul cas. Des mesures du taux de PTHrP chez des patients similaires seront nécessaires pour confirmer nos résultats.

**Conclusions:**

L’éventualité de taux élevés de PTHrP doit être envisagée chez les patients atteints de dysplasie rénale et chez lesquels on a autrement diagnostiqué une hypercalcémie.

## What was known before

Hypercalcaemia, while otherwise rare in infants, is sometimes seen in infants with congenital anomalies of the kidneys and the urinary tract (CAKUT). Only a small fraction of these patients have signs of a vitamin D overdose.

## What this adds

Our case report suggests that hypercalcaemia may result from the overproduction of PTHrP in patients with CAKUT and adequately suppressed PTH. Our case also suggests alternative approaches to maintain normocalcaemia with hydration, furosemide, and calcitonin, rather than bisphosphonate.

## Background

**Figure 1:**
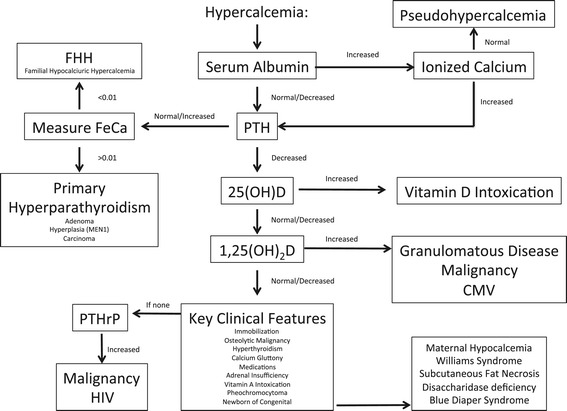
Diagnostic and treatment algorithm for hypercalcaemia in neonates and infants (adapted from Lietman et al. [1]).

Severe hypercalcaemia is a rare but clinically significant condition in infancy and childhood. Symptoms and complications may include stomach upset, nausea, vomiting and constipation, confusion, lethargy and fatigue, and calcification of the kidney with subsequent dehydration and excessive thirst. The differential diagnosis for hypercalcaemia in infancy and childhood includes: parathyroid hormone (PTH)-dependent anomalies, including MEN syndromes I and II and Jansen’s metaphyseal chondrodysplasia; calcium-sensing receptor disorders such as with severe neonatal hyperparathyroidism or familial hypocalciuric hypercalcaemia (a rare genetic disorder); vitamin D-related anomalies including vitamin D intoxication, Williams syndrome, and granulomatous diseases; and disorders related to vitamin A intoxication or immobilization (Figure 1) [1]. Hypercalcaemia may also be the product of a malignancy, often mediated by parathyroid hormone-related protein (PTHrP) [2,3]. PTHrP is homologous in structure to PTH. It works both in a paracrine and autocrine fashion and can be found in epithelial and mesenchymal tissues, endocrine glands, and the central nervous system [4]. Although rare, benign tumours can also secrete PTHrP [5-7]. This phenomenon was recently reported in benign mesoblastic nephroma [8]. Grob et al. provided another example with their recent report of PTHrP-related hypercalcaemia in an infant with renal dysplasia in the absence of a tumour [9]. Here we report another case of hypercalcaemia associated with elevated PTHrP levels in the context of known renal dysplasia in an infant. Diagnostic workup and management are discussed. Explicit written consent for this case report was obtained.

### Case report

**Figure 2:**
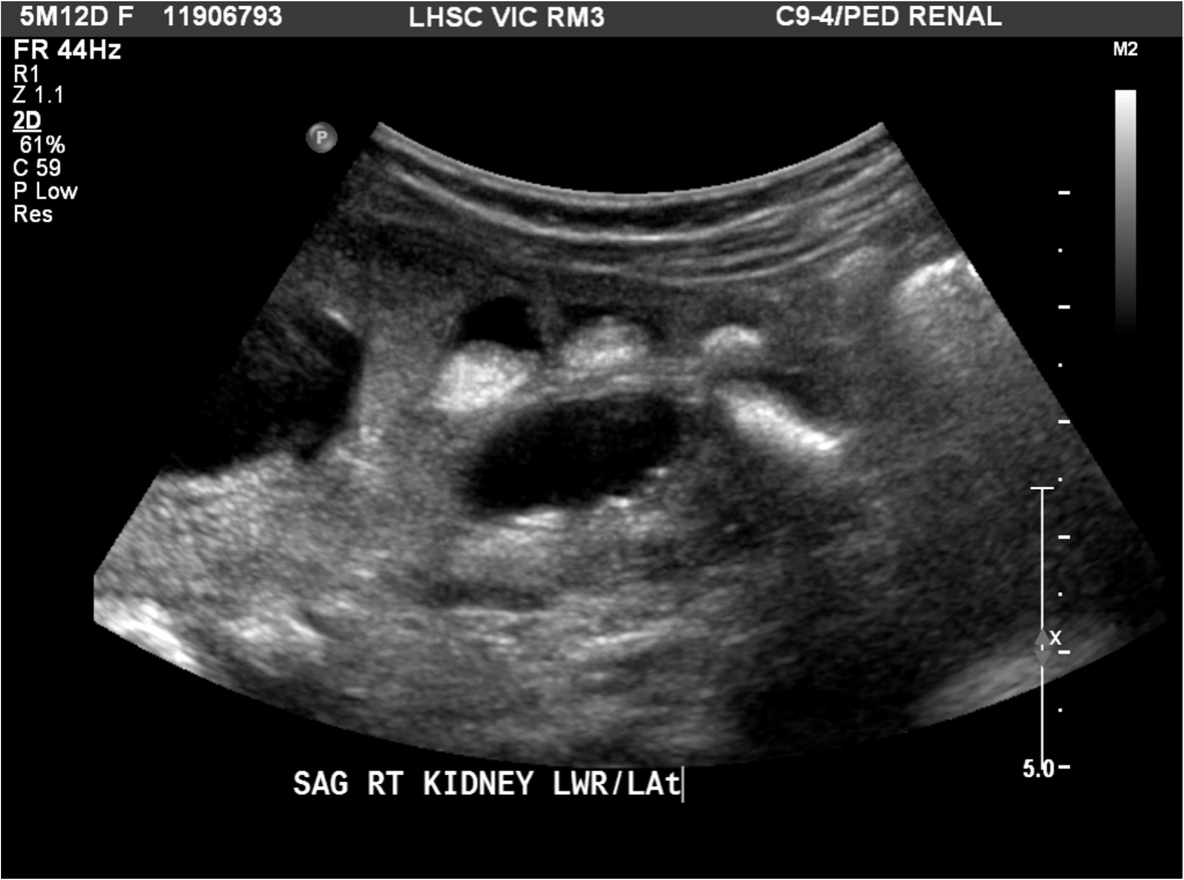
Representative section of the right kidney showing multiple echoic structures in the calices without shadowing.

A 5-month-old Caucasian female infant with congenital anomalies of the kidneys and urinary tract (CAKUT) presented to our centre with a history of emesis, decreased oral intake, and lethargy. She had been born at 40 weeks and 4 days after an uncomplicated pregnancy with a birth weight of 4010 grams to unrelated parents. In a previous examination, she was found to have bilateral renal duplication and bilateral ureteroceles corresponding to the upper moieties, with grade 5 vesicoureteric reflux in both lower moieties. The ureteroceles were managed with bilateral ureterocele incisions. A renal ultrasound found normally sized (6 cm long) kidneys with bilateral renal duplication, bilateral tortuous hydroureter, and thin renal cortex with increased cortical echogenicity and small cysts (Figure 2). Her serum creatinine peaked at 117 μmol/L after birth. At 3 months of age, her cystatin C-estimated glomular filtration rate (eGFR) [10,11] was 51 mL/min/1.73 m^2^ and at 5 months it rose to 64 mL/min/1.73 m^2^, indicating stage 2 chronic kidney disease (CKD). The patient was not on any medication at the time of admission. Her weight dropped from 5180 grams at admission to 4890 grams one week later, a loss of 6%. On examination, she exhibited signs of dehydration with sunken fontanels and dry mucous membranes. She was afebrile. Blood work showed a calcium level of 4.98 mmol/L (reference interval (RI) 2.24-2.74 mmol/L), phosphate 0.83 mmol/L (RI 1.30 to 2.60 mmol/L), creatinine 63 μmol/L (RI < 53 umol/L), urea 13.7 mmol/L (RI < 7.0 mmol/L) and bicarbonate 15 mmol/L (RI 22–29 mmol/L). Her serum sodium was 134 mmol/L and her fractional sodium excretion was 20.06%, suggesting some sodium wasting, which is frequently seen in patients with renal dysplasia.

### Diagnostic work-up

An extensive investigation was undertaken to determine the aetiology of this child’s hypercalcaemia. Urine testing showed significant hypercalciuria with elevated Uca/cr ratios of up to 9.8 (normal <0.6) mmol/mmol. A renal ultrasound showed multiple echogenic structures in both kidneys and bilateral calcifications of the renal pyramids (Figure 1). PTH was adequately suppressed at 0.4 (normal 1.6-6.9) pmol/L, thereby ruling out primary hyperparathyroidism. Secondary and tertiary hyperparathyroidism were highly unlikely in view of the mild decrease in GFR (CKD 2). TSH, Free T4, and AM cortisol were normal, ruling out hyperthyroidism and adrenal insufficiency. There was no clinical evidence of acromegaly. Pheochromocytoma was ruled out given that the patient was normotensive, and HMA and VMA levels were normal. The patient’s 25-vitamin D level was normal at 140 pmol/L (RI 75 to 225 nmol/L), while her 1,25 dihydroxy-vitamin D level was unexpectedly low at 10 pmol/L, ruling out Vitamin D excess. Vitamin A levels were low, also ruling out vitamin A intoxication. Normal or low ACE levels indicated that sarcoidosis was unlikely. A normal chest x-ray revealed no radiological evidence of tuberculosis. Finally, the patient had low calcitriol levels were low, which is inconsistent with granulomatous disease. Given that the infant’s calcium levels had previously been normal and she did not have a family history of familial hypocalciuric hypercalcaemia this was an unlikely diagnosis. The patient was not on any medications known to cause hypercalcaemia. There was also no evidence of rhabdomyolysis. Bone lysis and immobilization-related high bone turnover was ruled out with the patient’s normal alkaline phosphatase results and skull and chest x-rays negative, which were negative for bony changes. Given that all other potential causes for hypercalcaemia had been ruled out, our haematology oncology colleagues were consulted to rule out malignancy-related humoral hypercalcaemia. A normal CBC and tumour lysis profile ruled out leukemia, the most common cause of malignancy-related hypercalcaemia in this age group. There was no clinical or radiological evidence of lymphoma and normal urine levels of catecholamine breakdown products ruled out neuroblastoma. An ultrasound showed no hepatosplenomegaly, and a whole-body MRI revealed no radiological evidence of malignancy. Malignancy-related humoral hypercalcaemia was therefore an unlikely aetiology. The patient’s PTHrP level was, however, found to be elevated at 3.8 (normal <2, Mayo Medical Laboratories) pmol/L [8], and phosphate wasting (TmP/GFR was 0.59, reference range for this age 1.15-2.60) [12] suggested PTHrP-related hypercalcaemia.

### Treatment

**Figure 3:**
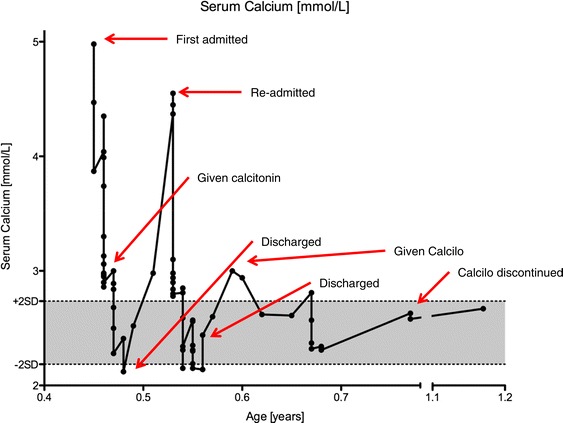
Serum calcium concentrations from initial admission to last follow-up.

Initial treatment consisted of aggressive rehydration with intravenous fluids (Ringers lactate, 20 mL/kg, repeated three times). Her calcium levels did not initially respond, and she was given intravenous furosemide (1 mg/kg intravenously, possible up to 10 mg/kg/day) and calcitonin (initially 2 units/kg subcutaneously, then 4 units/kg/day every 24 hours, could be given every 12 hours) with good effect (serum calcium dropped to 2.14 mmol/L in 48 hours). Her calcium levels remained stable with all interventions withdrawn for several days prior to her discharge. Her calcium level at time of discharge was 2.12 mmol/L. Unfortunately, she was readmitted for recurrent hypercalcaemia one month after her initial discharge with a calcium level of 4.37 mmol/L. She was once again treated with intravenous fluids, Lasix, and calcitonin again. Her calcium level at time of discharge was 2.44 mmol/L (Figure 3). A further one month later, outpatient laboratory testing revealed an elevated calcium level of 3.0 mmol/L, prompting the introduction of CalciLo (a low-calcium, vitamin D-free infant formula that contains 1/10th the calcium concentration of breast milk). CalciLo was discontinued 6 months later, and her calcium levels have remained stable after 6 months of follow-up visits. Table 1 provides the laboratory findings at presentation and at last follow-up.

**Table 1:** **Patient serum and urine parameters at presentation and last follow-up**

**Serum or urine parameter**	**At presentation**	**At last follow-up**	**Age-appropriate reference interval**
Serum creatinine	63 μmol/L	34 μmol/L	<53 μmol/L
Cystatin C eGFR	51 mL/min/1.73 m^2^	89 mL/min/1.73 m^2^	>90 mL/min/1.73 m^2^
Serum calcium	4.98 mmol/L	2.67 mmol/L	2.24-2.74 mmol/L
Serum urea	13.7 mmol/L	6.1 mmol/L	<8.0 mmol/L
Serum bicarbonate	15 mmol/L	21 mmol/L	22-29 mmol/L
Serum sodium	134 mmol/L	139 mmol/L	135-145 mmol/L
Fractional sodium excretion	20.06%	n.d.	N/A
Urine calcium	2.93 mmol/L	1.45 mmol/L	N/A
Uca/cr ratio	9.8 mmol/mmol	0.822 mmol/mmol	<1 at 6 months, <0.6 at 2 years
PTH	0.4 pmol/L	3.3 pmol/L	1.6-6.9 pmol/L
25-vitamin D	140 nmol/L	94 nmol/L	75-225 nmol/L
1,25 dihydroxy-vitamin D	<10 pmol/L	n.d.	39-193 pmol/L
Vitamin A	0.7 μmol/L	n.d.	1.2-2.8 μmol/L
Alkaline phosphatase	206 U/L	296 U/L	<449 U/L
PTHrP	3.8 pmol/L	n.d.	<2 pmol/L
Phosphate	0.83 mmol/L	1.83 mmol/L	1.3-2.6 mmol/L

## Discussion

This report highlights a case of severe hypercalcaemia in an infant with CAKUT, CKD stage II, and ultrasound findings suggesting renal dysplasia. The sodium wasting seen at admission is indicative of renal dysplasia. The extensive workup performed left PTHrP-related hypercalcaemia as the most probable aetiology. The case was interesting as hypercalcaemia is uncommon in infancy, and PTHrP-associated hypercalcaemia without malignancy or benign renal mass is rarely described. To the best of our knowledge, there has only been one published case report describing a similar patient [9], although hypercalcaemia has been reported in as many as 15% of patients with CAKUT [13]. Our case differed from the 2 patients described by Srivastava et al., where benign congenital mesobastic nephroma caused substantial PTHrP elevations [8]. Our case is similar to that described by Grob et al., who described an infant with stage III chronic kidney failure secondary to multicystic dysplastic kidney disease who presented with severe hypercalcaemia, suppressed PTH, and elevated PTH-related peptide at 3 months of life [9]. Malignancy was also ruled out in that case and unlike our patient, the patient was treated with bisphosphonates twice, resulting in a successful though delayed response.

PTHrP is a pleiotropic factor with multiple physiological functions in morphogenesis, cell proliferation, differentiation, apoptosis, and calcium homeostasis. In the kidney, PTHrP is abundantly expressed and upregulated in various experimental nephropathies, showing growth-modulatory and pro-inflammatory properties [14]. Despite the widespread production of PTHrP in the normal tissues of healthy individuals, the concentration of the protein is below the detectable limit of current assays (<0.2 pmol/L in the N-terminal(1–86)-region-specific PTHrP radioassay) [15], suggesting that PTHrP normally functions locally in an autocrine or paracrine manner. PTHrP also has a proliferative effect on both glomerular mesangial cells and tubular epithelial cells [16]. Increases in the expression of PTHrP have been observed in several experimental models of nephropathies, suggesting that PTHrP upregulation is a common event associated with the mechanism of renal injury and repair [16]. Our case report suggests that hypercalcaemia may result from the overproduction of PTHrP in patients with renal injury and adequately suppressed PTH.

Our case is important because hypercalcaemia in children with CAKUT is relatively common and may be unrelated to vitamin D therapy [13]. The low vitamin D level is actually consistent with the diagnosis, as a low level is protective and often seen in patients with hyperparathyroidism or (as in our case) PTHrP elevation. We acknowledge that availability of PTHrP testing is limited and the several week turn around time of several weeks is impractical for the acute management. Our case may also suggest alternative approaches to maintain normocalcaemia with hydration, furosemide, and calcitonin. Alternatives would be beneficial because bisphosphonate therapy is not well-established in infants, may be associated with severe skeletal toxicity [17], and could perhaps be reserved for calcitonin-resistant cases. In Al-Kalbani’s series, 15 of 99 infants with renal dysplasia had hypercalcaemia, and patients with obstructive uropathy, as was true in our case, were most commonly affected [13]. In Al-Kalbani’s series, the mean Schwartz eGFR was 57 mL/min/1.73 m^2^, similar to our patient, but but the range was wide. Only 30% of the patients in that series had an elevated PTH, while the others were similar to our patient [13]. More importantly, the mean duration of the hypercalcaemia was 5.2 months in Al-Akalbani’s series [13], which may significantly contribute to the substantial risk of vascular calcifications, a potentially life limiting sequela recognized in children with CKD [18]. The use of intravenous hydration, furosemide, and calcitonin allow for a rapid normalization of hypercalcaemia [19], and the use of a low-calcium diet alone may be advantageous in maintaining a more long-term normocalcaemia over bisphosphonates.

## Conclusions

We conclude that hypercalcaemia may result from the overproduction of PTHrP in patients with CAKUT and adequately suppressed PTH, and that hypercalcaemia secondary to PTHrP in children with CAKUT, particularly when they have an element of obstruction, may be under-recognized. Investigations should include PTH, and if this is adequately suppressed, measuring PTHrP should be considered. Intravenous hydration, furosemide, and calcitonin facilitate a rapid normalization of the serum calcium, and normocalcaemia may be maintained with CalciLo rather than with bisphosphonates.

## Abbreviations

CAKUT: Congenital anomalies of the kidneys and urinary tract
eGFR: Estimated glomerular filtration rate
GFR: Glomerular filtration rate
PTH: Parathyroid hormone
PTHrP: PTH-related peptide
Uca/cr: Urinary calcium/creatinine
